# Dietary Habits and Nutritional Knowledge of Pregnant Women: The Importance of Nutrition Education

**DOI:** 10.3390/foods13193189

**Published:** 2024-10-08

**Authors:** María Josefa Olloqui-Mundet, María del Mar Cavia, Sara R. Alonso-Torre, Celia Carrillo

**Affiliations:** Nutrición y Bromatología, Facultad de Ciencias, Universidad de Burgos, E-09001 Burgos, Spainmmcavia@ubu.es (M.d.M.C.); salonso@ubu.es (S.R.A.-T.)

**Keywords:** pregnancy, Mediterranean diet, nutritional knowledge, nutrition education

## Abstract

A good diet during pregnancy is associated with improvements to maternal and fetal health. Nevertheless, excessive weight gain during pregnancy has been reported in several studies. The aim of this review is to determine the dietary habits of pregnant women (measured according to their degree of adherence to the Mediterranean diet, a reference in dietary quality), their knowledge of food and nutrition, and their perceptions of the nutritional education received during their pregnancy, in order to detect intervention needs within that group. The bibliographic search was conducted on three databases (Medline, PubMed central, and Web of Science), using the keywords “pregnancy”, “Mediterranean diet”, “nutrition knowledge”, “nutritional education”, and some synonyms. The final selection included 68 original articles. The available evidence indicated that, although pregnant women were aware of the importance of nutrition during pregnancy, their habits showed some room for improvement in terms of diet and physical exercise. Lack of adequate advice was the main barrier to the practice of healthy habits that pregnant women encountered; they considered that the information they received during pregnancy follow-up visits was inadequate. However, the success of interventions within different countries is a source of hope for well-structured nutrition education throughout pregnancy. The lack of nutrition-related knowledge among pregnant women could be originally related to poorly planned nutrition education from healthcare providers. Research focused on the consultations with these healthcare providers could be of help when proposing strategies to improve the content, the depth, and the duration of nutrition education sessions. It should, in any case, be noted that the available evidence in this field is limited to certain geographical origins. Therefore, research that uncovers evidence in different countries will be useful for learning about the factors that condition the habits of pregnant women and, in that way, guide strategies for the improvement of the health of expectant mothers during that stage in their lives.

## 1. Introduction

Maternal health is defined as the health of women from the preconception stage, through pregnancy and childbirth, to the postpartum period. During pregnancy, energy and nutritional needs increase (especially in the second and third trimesters) and, to meet those needs, pregnant women must have access to quality nutritious food in sufficient quantities. Thus, maternal nutritional status is a key determinant of the health of pregnant women [1]. Good nutritional status during pregnancy is associated with better maternal and fetal health and better perinatal outcomes. In that regard, the general recommendation is to follow a healthy balanced diet [2].

The Mediterranean diet is among the most widely studied dietary patterns in relation to its effects on health. It is characterized by a high intake of fruits, vegetables, whole grains, legumes, fish, and nuts, and by a moderate intake of dairy products, eggs, red meat, and wine. Nutritionally speaking, the diet is low in saturated fats and high in fiber, antioxidants, and mono- and polyunsaturated fatty acids. The potential of this dietary pattern for pathologies such as, among others, type 2 diabetes, cardiovascular diseases, different types of cancer, and neurological diseases, has been confirmed in several reviews and meta-analyses [3,4]. Its benefits make it especially interesting during pregnancy, since good adherence to this dietary pattern during pregnancy is related to beneficial effects on fetal growth, the prevention of neural tube defects, prematurity, asthma and childhood allergies, maternal and fetal body weight, different fetal cardiometabolic markers, and infant skin diseases, among others [5]. Recent studies have also confirmed that stricter adherence to the Mediterranean diet during pregnancy is associated with a greater diversity in the intestinal microbiota of pregnant woman, which could have beneficial effects for the mother, as well as for the colonization of the intestinal microbiota of the baby after birth [6].

Nevertheless, despite the supporting evidence for the benefits of this dietary pattern, there is little adherence to healthy eating recommendations during pregnancy [7], and low levels of nutritional knowledge have been observed among pregnant women [8]. Raising awareness of the health risks to mother and fetus associated with inadequate gestational habits could have a positive impact on these habits [9]. Moreover, gestation is an ideal time to promote healthy habits, because pregnant women are highly motivated to do their best for their children. In that regard, dietary counseling has been identified as the intervention that is most likely to improve dietary and nutritional knowledge, as well as lifestyle [10]. The World Health Organization (WHO) has highlighted the importance of health professionals providing nutrition education during each prenatal visit to maximize pregnancy success [11].

Considering that the midwife is the health professional who is best positioned to educate pregnant woman in nutrition in different counties, Olloqui-Mundet et al. [12] recently reviewed the role of the midwife in nutrition education, midwifery attitudes, and any relevant training. In that regard, it should in general terms be noted that midwives recognize their role as health educators and, in practice, talk about nutrition to pregnant woman. Even so, inadequate weight gain during gestation has been confirmed in several studies on populations of different origin [13,14,15,16,17,18], which would call into question the quality of the nutrition education that has been received.

This study is focused on pregnant women of varied origin, with the aim of providing an updated review (taking evidence from the past 10 years) of (i) the dietary habits of pregnant women; (ii) their knowledge of food and nutrition; and (iii) their perceptions of the nutrition education that they received during pregnancy, in order to detect intervention needs within the group.

## 2. Materials and Methods

Medline, PubMed Central, and Web of Science were the electronic databases selected for conducting the literature search. The key search terms were “Mediterranean diet”, “nutritional knowledge”, “nutrition education”, “pregnancy”, and corresponding synonyms, with their presence limited to title and abstract to refine the search. Those terms were combined with the corresponding Boolean operators. Additionally, the bibliographic references of the articles that the search returned were also inspected to identify further studies.

The articles for inclusion in the review had to have been published within the past ten years. The inclusion criteria were: original papers that were written in either English or Spanish on the following subjects: (i) dietary habits (with a special focus on adherence to the Mediterranean diet, considered a benchmark in dietary quality) during pregnancy; (ii) nutritional knowledge among pregnant women; and (iii) nutritional education received during pregnancy.

Studies in which the topics under review were not addressed, brief communications, editorials and reviews, and papers on specific health problems among pregnant women were excluded from the review.

The search returned a total of 775 articles. A first reading of the titles and abstracts led us to eliminate 666 articles. Once the full texts of the remaining manuscripts had been examined, 68 original papers that matched the inclusion criteria formed the final literature review.

## 3. Results and Discussion

The studies included in the review were carried out in different countries: Australia (ten studies), Bangladesh (three studies), Brazil (one study), Canada (one study), Congo (one study), Croatia (one study), Czech Republic (one study), Egypt (three studies), Ethiopia (six studies), France (one study), Guadeloupe (one study), Greece (one study), Indonesia (one study), Iran (two studies), Italy (two studies), Japan (one study), Jordan (one study), Kenya (one study), Lebanon (one study), Malawi (one study), Malaysia (one study), Norway (one study), Pakistan (three studies), Poland (one study), Portugal (one study), Romania (one study), Spain (ten studies), Tanzania (one study), Turkey (two studies), Uganda (one study), the United Kingdom (two studies) and the USA (four studies).

A summary of the 68 selected articles is presented in Table 1.

This section is structured into three blocks, in accordance with the objectives of the review. First, the dietary habits of pregnant women will be discussed, followed by their knowledge of food and nutrition and, finally, the nutrition education they received during pregnancy.

### 3.1. Dietary Habits during Pregnancy

Most studies on the degree of adherence of pregnant women to the Mediterranean dietary pattern were conducted in countries around the Mediterranean basin. In that regard, most were Spanish, although contradictory results were observed in some of them. While Peraita-Costa et al. [19] observed high degrees of adherence among the majority of pregnant women in their sample, four studies found moderate adherence [20,21,22,23] and four studies found low adherence [24,25,26,27]. Most of the Greek pregnant women presented high adherence to the Mediterranean dietary pattern [28] and similar results were observed among the Italian women evaluated by De Giuseppe et al. [29], although Di Renzo et al. [30] found medium adherence among pregnant women of the same origin. Moderate to low adherence was observed among Croatian [31] and Portuguese [32] pregnant women.

In relation to studies carried out in non-Mediterranean countries, the highest degree of adherence was observed among Australian pregnant women [33], where 75% of the respondents showed moderate to high adherence to the Mediterranean dietary pattern. The majority of the pregnant women from Guadeloupe (French West Indies) and the USA had moderate-to-low adherence [34,35], and low adherence to the Mediterranean diet was found among Japanese pregnant women [36].

Thus, it appears that the geographical origin of a population is insufficient to guarantee dietary habits specific to the region. However, the diversity of tools used to assess the degree of adherence to the Mediterranean pattern among the studies under review makes it difficult to compare them.

Several authors have investigated the factors that influence the levels of adherence to the Mediterranean dietary pattern among pregnant women. In that regard, a higher degree of adherence was observed among Caucasian women [28], older women [28,37], those of high socioeconomic status and with higher education qualifications [37], as well as those with an active lifestyle, [33] and who consumed more fish, vegetables, and cereals [38]. On the other hand, younger age [20,39] and low socioeconomic and/or educational levels [39], lower fish consumption [24], higher consumption of processed foods [37], smoking [20,30,39,40], and alcohol consumption [21] were associated with low levels of adherence to the Mediterranean dietary pattern. Similarly, moving away from the Mediterranean dietary pattern is also associated with inactive lifestyles [39], with a high Body Mass Index (BMI) [33], and with lower consumption levels of folic acid supplements [20,24] and iron [24].

Changes in habits associated with the gestational stage have been evaluated in several studies. On that point, some authors noted that such changes even begin during the pregestational period. Portuguese women seeking to become pregnant increased their consumption of vegetables, fruits, nuts, and legumes, without reducing their intake of other foods found in the Mediterranean diet [32]. However, Tranidou et al. [28] observed that Greek women seeking pregnancy took no folic acid supplements, consumed alcohol, and in general only complied with the recommendations for dairy consumption. During the gestational stage itself, improvements in the habits of pregnant women of different origins (American, Australian, Croatian, Greek, Italian, and Spanish) were observed in various studies [23,28,30,32,41,42,43,44]. The most common changes consisted of avoiding foods with risks for pregnant women (such as unpasteurized dairy products, raw or smoked meats and fish, and fish with a high mercury content), taking supplements [31], and increasing consumption of fresh fruit, vegetables, dairy products, nuts, legumes, and fiber-rich foods [28,30,41]. Similarly, pregnant women often make changes in their habits to cope with the gastrointestinal problems of pregnancy [43]. In developing countries, those changes are at a different level. So, Ethiopian pregnant women [45] added at least one meal during pregnancy, met the recommendations for daily consumption of meat and legumes as a source of protein, green leafy vegetables, and dairy products, reduced caffeine consumption, and made massive use of iodized salt, in line with the country’s recommendations for pregnancy.

Nevertheless, this sort of improvement in dietary habit can never be extended to all pregnant women. Different authors observed increases in the consumption of unhealthy foods during pregnancy among Croatian, Greek, and Spanish pregnant women [21,28,31]. Surprisingly, a high proportion of women abandoned exercise when they become pregnant, despite the benefits associated with an active life during pregnancy. Specifically, 44% of the English pregnant women surveyed in the study by Swift et al. [43] admitted to exercising less after they had become pregnant. In addition, it may be noted that the improvements in dietary habits among the Australian pregnant women surveyed in the work of Lee et al. [46] were temporary and were rarely maintained over time.

### 3.2. Pregnant Women’s Knowledge of Food and Nutrition

In some studies, pregnant women’s overall knowledge of food and nutrition has been quantified through questionnaires designed for that purpose. For example, Jordanian pregnant women showed good nutritional knowledge, with an average score of 14.6 out of 20 [47]. Lebanese pregnant women also demonstrated adequate knowledge (mean score of 27 out of 41) [48]. In Bangladesh, 40.4% of pregnant women administered a questionnaire [49] gave the right answers to 13 or more questions out of the 15; 26.7% correctly answered 10–12 items, 20.2% gave the right answers to 6–9 items, and 12.4% correctly answered fewer than 6 items (cut-off points were excellent, good, moderate, and poor, respectively). Akhtar et al. [50] reported that half of the Pakistani pregnant women surveyed in their study had good knowledge (more than 75% out of a total of 30 questions; a score of less than 30% correct was considered poor knowledge). Razzaq et al. [51] found similar results in the study of pregnant women of the same origin (47.5% had good knowledge and 40% excellent knowledge). Likewise, Ikhsan et al. [52] found good levels of knowledge, although those authors never detailed the cut-off points for the measurement tool used in their study of pregnant women in Malaysia. Lower levels of knowledge were observed among pregnant women from Australia [46], where none reached 80% of the correct scores (mean score 34.5 out of 76); Egypt (48) (53 hits out of 146 items); Ethiopia [53], with a mean score of 4.1 out of 8; and the USA [42] (mean score 30 out of 57). Even so, the lack of uniformity and measurement scale validation meant that it is difficult to compare the results. Likewise, the construct “knowledge of food and nutrition” may have several associated dimensions, the analysis of which could be of great interest in this case, in order to assess the fields where pregnant women have the greatest training needs. So, the knowledge of pregnant women in relation to different topics is discussed below.

Various authors observed that pregnant women of diverse origin were aware of the importance of nutrition in pregnancy for the health of the mother and fetus [47,54]. The pregnant women knew that nutritional requirements increased during pregnancy [49,50] and that it was necessary to increase food intake [45,55], although some of them were unaware of which food groups should be promoted [41], or their recommended serving sizes [56].

In relation to nutritional requirements, it was observed in several studies that pregnant women of different origin (Australian, Czech, and North American women) [42,57,58] were mostly aware of the importance of folic acid supplementation during pregnancy. However, some of them were unaware of the recommended dosage, the ideal time to start supplementation, the reasons for the advisability of supplementation, and the dietary sources of folate [42,57]. In general terms, pregnant women have an even more limited knowledge of iodine [42,57,58,59], although Abu-Baker et al. [47] observed that Jordanian pregnant women were able to identify iodine-rich foods. Pregnant women from Pakistan and Jordan [47,50] were aware of the importance of iron during pregnancy, its requirements, as well as factors that activate and inhibit its absorption, such as vitamin C and tea or coffee, respectively. However, only one in three Ethiopian pregnant women knew that meat, liver, and fish were good sources of iron [45]. It was also confirmed in other studies that pregnant women were aware of the importance both of calcium [57] (although some were not able to identify their calcium requirements) [50] and of vitamin A during their pregnancy, as well as being aware of the risks associated with excesses and deficiencies of that vitamin [58,60]. Interestingly, although Czech pregnant women were aware of the increased omega-3 fatty acid requirements during gestation, Bookari et al. [58] noted that only 36% of Australian pregnant women surveyed were aware of the importance of those fatty acids for the development of the fetal retina and nervous system. There is controversy regarding macronutrient knowledge among pregnant women. Studies among Czech [57] and Jordanian [47] pregnant women observed that they were aware of where to find carbohydrates and proteins, whereas Pakistani pregnant women [50] were unaware of their needs for those nutrients, and Australian women hardly knew the carbohydrate contents of common foods [56]. In contrast, Congolese pregnant women had some limited knowledge of nutrients in general and where to find them [55].

Regarding the consumption recommendations of the different food groups, it should be noted that Bookari et al. [61,62] observed a great lack of knowledge of the Australian dietary guidelines among pregnant women in that country and that, among those who were aware of their existence, few possessed detailed knowledge [61]. It was nevertheless confirmed in studies on pregnant women of different origins (Egyptian, Australian, Tanzanian, or Czech) that they were aware of the consumption recommendations for some isolated food groups such as fruits and vegetables [41,60,63], dairy products, meat, fatty foods [41,63], and fish [57]. Wise et al. [44] observed that, although North American adolescent pregnant women had a general knowledge of healthy foods, they overestimated the power of vitamin supplements to compensate for unhealthy food intake.

Regarding other issues of importance to pregnancy, while Australian pregnant women were aware of the importance of physical activity during pregnancy, as well as of the corresponding recommendations [54], Swift et al. [43] observed that British pregnant women were unaware of the guidelines on physical activity during pregnancy. It was underlined in various other studies that the general knowledge of pregnant women on food safety issues was acceptable [58], that they knew which foods to avoid during pregnancy [45], and that they were able to identify those with a high risk of listeria [57,58] or salmonella [57]. Similarly, most of them knew how to manage gastrointestinal discomfort and how to remedy pregnancy constipation [58]. Yuste Gómez et al. [39] observed that Spanish pregnant women who developed diabetes during pregnancy had inadequate knowledge of the corresponding dietary guidelines; for example, they limited their consumption of fruit due to its sugar content, thus complicating the supply of vitamins and minerals that are essential in pregnancy.

Finally, several authors studied the knowledge of pregnant women on one of the most controversial topics during that stage of life: weight gain and associated recommendations. So, Australian pregnant women considered it to be a very important issue [61], yet Spanish women were unaware of the importance of healthy weight gain in pregnancy for the health of the developing fetus [39]. Studies conducted with pregnant women in Australia [41,61], England [37], Iran [64], Czech Republic [57], and the United States [65] indicated that they were unaware of the recommendations on gestational weight gain, although Abu-Baker et al. [47] observed that 60% of Jordanian pregnant women were aware of the healthy weight gain range when pregnancy begins in a situation of normal weight. Interestingly, overweight/obese women overestimated the range of healthy weight gain [61]. Notably, Al Wattar et al. [37] observed that English pregnant women were unaware of the recommended guidelines for managing healthy weight gain.

Several studies have investigated the factors that have influenced knowledge of food and nutrition among pregnant women. In that sense, better nutritional knowledge has been observed among older pregnant women, with higher educational and/or socioeconomic levels, of white race, and married status [41,49,50,52,57,62,66,67]. Pregnant women with training in nutrition or another health field had, in general, better knowledge levels [58]. The number of pregnancies [49] was another variable that was also associated with greater knowledge, with better results in primiparous (perhaps they had more time for training or they perceived a greater degree of responsibility towards the unknown) [57] and secundiparous women [58]. Similarly, pregnant women who planned their pregnancy, those in the first trimester of gestation, and those who suffered some health problem during pregnancy, had better knowledge [58]. Although Papežová et al. [57] observed worse knowledge among Czech pregnant women with extreme BMI (morbid obesity and underweight), Azad et al. [49] found no such association among Bangladeshi pregnant women.

Interestingly, judging from the findings of studies conducted with Australian [58] and Turkish [66] pregnant women, no association was found between the knowledge of pregnant women and the fact of either having received nutritional education during pregnancy [66] or having had access to a nutritionist [58]. However, two studies on pregnant women in Ethiopia found better dietary knowledge and habits among those who had attended prenatal consultations [53,68]. Similarly, the association between the level of knowledge and the habits of pregnant women has been confirmed in several studies [51,66,68], although Ikhsan et al. [52] observed that pregnant women in Malaysia, despite being aware of the dietary recommendations and having a good attitude towards them, never put them into practice. Something similar occurred among Tanzanian pregnant women, where no association between knowledge and habits was found [60]. Thus, nutritional knowledge, it appears, does not always imply adequate dietary habits. Eating behavior is complex and there are many factors that contribute to food choices. For instance, food availability and both economic and cultural factors are clear determinants of dietary choices, which may be related to the differences observed between countries. In the specific case of folic acid supplementation, several authors confirmed the relationship between knowledge of this supplement for the prevention of fetal problems and its usage [66,69].

### 3.3. Nutrition Education during Pregnancy

Studies on Australian pregnant women indicated that they considered knowledge of food and nutrition important, and were interested in receiving advice on healthy eating in pregnancy [54,62]. In that regard, many pregnant women, especially first-time mothers, actively sought information as soon as they became aware of their pregnancy [70,71]. They usually obtained information from different sources, among which the Internet stood out because of its easy access, although it is the least reliable source [42,46,65,70,71,72]. Books, relatives, and friends, especially those with children [55,70,71], may also be mentioned, although pregnant Australians thought that information from nearby sources was untrustworthy unless the person was a health professional [70]. In fact, the midwife was the main source of information and the one that generated the most trust [45,46,55,60,71,73].

Pregnancy follow-up visits are an ideal time to provide nutrition education [58]. The vast majority of pregnant women reported their pregnancy to a healthcare professional and had their first follow-up visit in the second or third month of gestation [69]. In that regard, Norwegian and Australian pregnant women considered that nutritional education had taken place too late [58,72] and would have preferred to receive the nutritional information at the beginning of their pregnancy, as soon as they knew they were pregnant, and even at preconception visits [70]. Furthermore, nutritional information was not, in their opinion, systematically provided as part of pregnancy follow-up [72]. In fact, North American pregnant women with a previous pregnancy reported receiving little nutritional information in the current pregnancy, because healthcare providers assumed that they already knew it [65].

Pregnant women who felt that they had not received sufficient information on healthy eating were identified in several studies [54,65,72] and, unexpectedly, pregnant women in Australia and Ethiopia reported not having received any nutritional education during pregnancy [45,46]. Among those pregnant women who did receive some nutrition education as part of the pregnancy follow-up programs, this education varied in content, format, clarity, and appropriateness [70].

American, Australian, Czech, English, and Norwegian pregnant women defined the information received as too general and impractical [46,57,62,70,72], saying that it was mainly focused on either the prevention of foodborne infections (toxoplasma and listeria) or food supplements [43,70]. According to French and Australian pregnant women, information was too focused on weight and its frequent control, which caused them stress [61,71]. In total, 33% of the American pregnant women surveyed in the study by Downs et al. [65] received no advice on appropriate weight gain in pregnancy during their first follow-up visit, nor did the Australian pregnant women [70]. Australian pregnant women rated advice as inadequate because they said that the importance of different nutrients, the risks of nutrient deficiencies, and food sources of nutrients [70] were never discussed. They also felt that the midwife had not given them much more than they already knew about nutrition [58], but that the information was more solid in the case of women with a health problem or special condition [70].

In addition to verbal information, many pregnant women received published information from their midwife in the form of government agency brochures or leaflets, along with other materials to consult at home. Such material was usually given at the end of the visit, and its content was not discussed during the visit [42,58,70]. Surprisingly, few pregnant women confirmed that they had consulted the documentation, claiming that they already had sufficient information at the time they received it (around week eight of pregnancy) [58]. That justification is striking, taking into account that many of the pregnant women identified lack of knowledge as one of the main barriers to practicing a healthy diet [38,46]. Other frequently mentioned barriers were lack of culinary skills and time [57], a mixture of social, cultural (beliefs and traditions), and economic factors (extreme poverty limits accessibility to certain foods and implies diets that are insufficient in quantity and quality) [42,55], laziness, and personal and environmental preferences [70]. Among those barriers, the lack of adequate nutritional counseling by health personnel should be highlighted [70,71,74].

Several authors have evaluated the effectiveness of nutritional interventions among pregnant women within different countries. In Ethiopia, different interventions were carried out to improve dietary and nutritional habits and knowledge, with very satisfactory results. Wakwoya et al. [75] and Demilew et al. [76] observed reductions in maternal malnutrition rates of 11% and 16.7%, respectively, in the intervention group. Pregnant women who received nutrition education increased arm circumference by 1.8% [75]. In the study of Diddana et al. [77], pregnant women with good dietary habits increased from 56.5% to 84.1%, in line with the significant increase in their knowledge levels after the intervention (from 6.9 to 13.5 out of 15), and with their greater perception of the severity of malnutrition and of the benefits for mother and fetus of healthy eating. Experiences in Bangladesh had a positive impact on the weight gain of pregnant women, and managed to increase the number of meals per day of the pregnant woman, the variety of their diet, and the consumption of animal protein, which had positive impacts on the weight gain of pregnant women and newborns [78,79]. In the study by Chowdhury et al. [79], a 57% reduction in the risk of low birth weight was achieved. In addition, the interventions developed in Bangladesh achieved the early establishment of breastfeeding and its maintenance as a means of infant feeding [78], which proved an excellent measure to reduce neonatal mortality. In Kenya, an association was also found between the weight of newborns and the participation of pregnant women in nutritional education programs (a reduction from 6.7% to 2.5% in the prevalence of low birth weight). Similarly, the percentage of preterm births after the intervention and the prevalence of pica (caused by micronutrient deficiency, mainly iron) decreased [80]. In a nutritional intervention developed in Pakistan, it was possible to increase the number of pregnant women who reached the recommended intake of cereals and potatoes, vegetables, fruits, and dairy products [81]. The results of studies in Brazil [82], Egypt [83,84], England [37], Iran [85], Jordan [47], Lebanon [48], Malawi [86], Pakistan [81], and the USA [42] have corroborated significant improvements in knowledge, attitudes, and habits after the intervention. More adequate weight gain indices were also observed among pregnant women and fetuses in the intervention group.

**Table 1:** Key characteristics of the studies included in the review.

Country	Aim of the Study	Method	Sample Size	Response Rate	Reference
Australia	To research pregnant women’s knowledge of optimal gestational weight gain and recommended dietary approaches for weight management	Online self-completed survey for knowledge and dietary recommendations	326	NI/NA	Bookari et al., 2016 [58]
Australia	To research pregnant women’s knowledge of the Australian guide to healthy eating and dietary recommendations for maintaining a healthy pregnancy	A cross-sectional study. Multidimensional online survey based on validated and existing measures.	400	NI/NA	Bookari et al., 2016 [61]
Australia	To explore pregnant women’s diets in relation to the Australian guidelines for healthy eating; factors influencing adherence to the recommendations; and attitudes towards pregnancy-specific nutrition information.	A cross-sectional study using convenience sampling through an online link	388	NI/NA	Bookari et al., 2017 [62]
Australia	To explore Australian women’s experiences in gaining nutrition information during pregnancy	Individual in-depth semi-structured telephone interviews	26	39.8%	Bookari et al., 2017 [70]
Australia	To explore pregnant women’s carbohydrate and standard serve size knowledge	Online survey, including a modified carbohydrate knowledge questionnaire	186	NI/NA	Brown et al., 2021 [56]
Australia	To explore nutrition and physical activity behaviors of healthy and overweight pregnant women and their knowledge	Prospective observational study. Self-administered semi-quantitative survey for knowledge (items from previous validated questionnaire) and dietary behaviors	58	63%	de Jersey et al., 2013 [54]
Australia	To research pregnant iodine supplement intake and health knowledge	Self-administered questionnaire on third trimester	200	NI/NA	Martin & Savige 2014 [59]
Australia	To investigate the nutrition knowledge of pregnant women, their main sources of information, and changes to their diet during pregnancy	Web-based pregnancy nutrition knowledge questionnaire (76 items). Face and content validity assessed and piloted	114	69%	Lee et al., 2016 [41]
Australia	To explore the nutrition knowledge of pregnant women and how nutrition knowledge impacts food choices	Mixed methods study design: questionnaire and interviews	105	NI/NA	Lee et al., 2018 [46]
Australia	To investigate the effect of prepregnancy BMI in the association between dietary patterns and the risk of gestational diabetes mellitus and hypertensive disorders of pregnancy.	Self-administered questionnaires	3378	NI/NA	Schoenaker et al., 2016 [33]
Bangladesh	To research level of pregnant nutrition knowledge among mothers attending antenatal care	Semi-structured questionnaire (15 items)	356	NI/NA	Azad et al., 2021 [49]
Bangladesh	To explore the impact of nutrition education of pregnant women on birth weight in rural Bangladesh.	Nutrition education intervention (nutrition training, balanced diet, and cooking techniques)	779 (382 IG/397 CG)	NI/NA	Chowdhury et al., 2022 [79]
Bangladesh	To explore the effect of short-term nutrition education on weight gain in the third trimester of pregnancy, birth outcomes, and breastfeeding	Nutritional intervention	300 (150 IG/150 CG)	93.2%	Jahan et al., 2014 [78]
Brazil	To assess the effectiveness of a nutritional counseling intervention concerning unprocessed and minimally processed foods rather than ultra-processed products, and the practice of physical activity to prevent excessive gestational weight gain in overweight pregnant women	Two-armed, parallel, randomized controlled trial. Nutrition intervention. Interviews	350 (169 IG/166 CG)	88.4%	Sartorelli et al., 2023 [82]
Canada	To explore diet quality changes in pregnancy and maternal characteristics associated with trimester-specific diet quality	Nine 24 h dietary recalls (3 each trimester, 2 weekdays, and 1 weekend day) through a web-validated questionnaire	79	NI/NA	Savard et al., 2019 [38]
Congo	To investigate the dietary knowledge and practices of a pregnant woman	Qualitative. In-depth interviews and focus group	9	75%	Maykondo et al., 2022 [55]
Croatia	To explore adherence to the Mediterranean diet	Data from the “Croatian Islands Birth Cohort Study”. Mediterranean diet questionnaire	266	NI/NA	Auguštin et al., 2020 [31]
Czech Republic	To explore the level of pregnant nutritional knowledge	Anonymous self-administered paper-form questionnaire (40 items plus 5 Likert scales)	401	NI/NA	Papežová et al., 2023 [57]
Egypt	To investigate the effect of the Mediterranean diet on the in-utero body fat formation and cord leptin level of newborns	Intervention among obese women with dietetic counseling based on the Mediterranean diet	118 (57 IG/61 CG)	NI/NA	Abdou et al., 2020 [83]
Egypt	To investigate pregnant nutritional knowledge and behavior and to identify the factors influencing both	Comparative cross-sectional study on public/private hospitals. Interview questionnaire (revised and piloted) to assess nutrition knowledge and dietary behavior	300	NI/NA	Nasrallah et al., 2020 [63]
Egypt	To assess the effect of nutritional health education on changing knowledge, attitude, and practice towards healthy pregnancy	Intervention study (pre- and post-test). Individual face-to-face interviews and semi-structured questionnaire	135	NI/NA	Soliman et al., 2019 [84]
Ethiopia	To research dietary practice and associated factors among pregnant women	A cross-sectional study. Interviewer-administered questionnaire	618	100%	Abute et al., 2020 [68]
Ethiopia	To investigate the effect of intensive nutrition education and counseling on nutritional status during pregnancy	Intervention. Structured questionnaires through one-to-one interviews of the participants at their homes	645 (313 IG/332 CG)	NI/NA	Demilew et al., 2020 [76]
Ethiopia	To assess the effect of nutrition education on nutritional knowledge and dietary practice of pregnant women	Community-based cluster randomized control trial. Nutrition education intervention	138	NI/NA	Diddana et al., 2018 [77]
Ethiopia	To investigate the effect of intensive nutrition education and counseling on nutritional status during pregnancy	Two-arm parallel design cluster randomized controlled trial. Intervention: intensive nutrition education and counselling package	374 (185 IG/189 CG)	NI/NA	Wakwoya et al., 2023 [75]
Ethiopia	To investigate dietary practices and their determinants among pregnant women	Community-based cross-sectional study. Questionnaire pre-tested (demographics and knowledge, attitudes, and practices)	351	100%	Yalewdeg et al., 2020 [53]
Ethiopia	To explore the effect of nutrition education on the knowledge and practice of pregnant women during pregnancy	Pre- and post-intervention study	422 pre- and 406 post-intervention	NI/NA	Zelalem, et al., 2017 [45]
France	To evaluate nutrition concerns, beliefs, and attitudes of pregnant women and their nutrition-related information-seeking behavior	A qualitative study. Seven focus groups	40	NI/NA	Bianchi et al., 2016 [71]
Greece	To explore the relationship between adherence to the Mediterranean diet before conception and the risk of gestational diabetes mellitus	A prospective cohort study. Dietary assessment with a validated FFQ, and maternal anthropometrics recorded at the antenatal visit	743	NI/NA	Tranidou et al., 2023 [28]
Guadeloupe (French West Indies)	To assess the effect of adherence to the Mediterranean diet during pregnancy on fetal growth restrictions and preterm delivery.	Data from the “TIMOUN Mother–Child Cohort Study”	728	NI/NA	Saunders et al., 2014 [34]
Indonesia	To explore the effects of a health and nutrition educational intervention on maternal knowledge, attitudes, and practices.	Health and nutrition education monthly. Structured questionnaires pre- and post-intervention	252 (127 IG/125 CG)	NI/NA	Wijaya-Erhardt et al., 2014 [73]
Iran	To explore the impact of educational programs with spousal participation on the optimal gestational weight gain	Intervention (3 groups). A: pregnant women received nutritional and physical activity education with their spouses, B: alone, and Control: did not receive	124	86.2%	Asiabar et al., 2018 [64]
Iran	To explore the effect of a nutrition educational program based on HBM compared with traditional education which recommended weight gain among pregnant women	A quasi-experimental (interventional) study. Questionnaire (83 items). Validity and reliability assessed	110 (54 IG/56 CG)	NI/NA	Mohebi et al., 2013 [85]
Italy	To retrospectively investigate the association between being small for gestational age, maternal adherence to the Mediterranean diet, lifestyle habits, and other risk factors during pregnancy	Data collected from medical records. Mediterranean diet adherence assessed through validated MEDI-LITE questionnaire	100	NI/NA	De Giuseppe et al., 2021 [29]
Italy	To investigate how a greater or lesser adherence to the Mediterranean diet influences specific parameters of mother and newborn	Anonymous online semi-structured questionnaire (79 questions) including a validated 14-item MEDAS	501	NI/NA	Di Renzo et al., 2022 [30]
Japan	To investigate the effect of adherence to the Mediterranean diet in pregnancy on the allergies of the offspring	Data collected on the Mediterranean diet during pregnancy and the incidence of allergies in offspring from the “Japan Environment and Children’s Study”	46,532 mother-newborn pairs	NI/NA	Nakano et al., 2023 [36]
Jordan	To explore the effect of health education on pregnant dietary knowledge levels and practices	Intervention. Structured questionnaire to assess dietary knowledge and practices before and after the intervention	195 (95 GI/100 CG)	92.7%	Abu-Baker et al., 2021 [47]
Kenya	To evaluate the effectiveness of home-based maternal nutritional counseling on nutritional outcomes, morbidity, breastfeeding, and infant feeding practices	Nutritional counselling intervention. Semi-structured questionnaires	1001 (480 IG/521 CG)	NI/NA	Nyamasege et al., 2019 [80]
Lebanon	To investigate the impact of nutrition education on the nutrition knowledge of pregnant women	Intervention. Pre- and post-nutrition knowledge test (41 items; true/false)	91	NI/NA	Chehade et al., 2023 [48]
Malawi	To investigate the effects of supplementary nutrition education and dietary counseling compared with routine antenatal care on pregnant nutrition knowledge, perceptions, and dietary habits	Intervention	195 (92 IG/103 CG)	NI/NA	Katenga-Kaunda et al., 2020 [86]
Malaysia	To research nutritional knowledge, attitude, and practice during pregnancy and the relationship with socio-demographic characteristics	A cross-sectional study. Questionnaire to assess knowledge (19 items), attitudes (14 items), and practices (13 items)	320	NI/NA	Ikhsan et al., 2018 [52]
Norway	To investigate experiences with nutrition-related information during routine antenatal care among women of different ethnical backgrounds	Individual interviews twice during pregnancy. Interviews followed semi-structured guides (pilot-tested).	17	NI/NA	Garnweidner et al., 2013 [72]
Pakistan	To explore pregnant nutritional knowledge about food intake during pregnancy and associations between education level and nutritional knowledge	A cross-sectional study	372	NI/NA	Akhtar et al., 2020 [50]
Pakistan	To research the effects of nutrition education intervention on dietary practices and the nutritional status of pregnant women	Quasi-experimental study. Intervention (two months). Pre- and post-counselling interviews	194	NI/NA	Kaleem et al., 2020 [81]
Pakistan	To explore pregnant nutritional knowledge and practices	Hospital-based cross-sectional study. Questionnaire	120	NI/NA	Razzaq et al., 2018 [51]
Poland	To investigate the nutritional behavior of pregnant women attending antenatal classes in comparison to non-attendees regarding the frequency of consumption of the main types of food	Questionnaire survey (28 multiple-choice items)	200	NI/NA	Lugowska et al., 2020 [40]
Portugal	To assess predictors of adherence to the Mediterranean diet from the first to second trimester of pregnancy	Prospective study. Socio-demographic and lifestyle characteristics assessed through a questionnaire. Food consumption assessed with a three-day food diary during first and second trimesters.	102	97.8%	Abreu et al., 2015 [32]
Romania	To assess the relationship between nutritional knowledge and the use of folic acid, iron, and multivitamin supplements during pregnancy, and the influence of socio-demographic factors and prenatal care	A cross-sectional study. Nutritional knowledge assessed using a standardized questionnaire (26 items) revised and validated in an interview	400	97.5%	Popa et al., 2013 [69]
Spain	To research the effect of adherence to a MedDiet pattern after 12 gestational weeks on maternal–fetal outcomes.	Post hoc analysis of the “St. Carlos Gestational Diabetes Mellitus Prevention Study”	874	58.2%	Assaf-Balut et al., 2018 [23]
Spain	To explore the relationship between maternal MedDiet adherence during pregnancy, anthropometric measures, and small-for-gestational-age at birth	A longitudinal population-based study analyzing data from healthy pregnant women from the ECLIPSES trial. Dietary assessment through FFQ	614 mother-newborn pairs	NI/NA	Díaz-López et al., 2022 [26]
Spain	To evaluate adherence to the Mediterranean diet and dietary guidelines, changes in diet during pregnancy and post-partum, and maternal factors associated with food consumption	Longitudinal study: clinical history, anthropometric measurements, lifestyle habits, and nutrition assessed at first, second, third trimester, and post-partum by interview using validated FFQ	Week 12: 793; week 24: 547; week 36: 465; postpartum: 418	NI/NA	Jardí et al., 2019 [21]
Spain	To evaluate whether adherence to the MedDiet during pregnancy induces health benefits for the offspring during the first two years of life	Nutritional intervention. Adherence to a healthy lifestyle assessed with the “Diabetes Nutrition and Complication Trial” questionnaire. Adherence to MedDiet assessed with MEDAS	703 mother-children (365 CG/338 IG)	80.4%	Melero et al., 2020 [27]
Spain	To evaluate adherence to the Mediterranean diet among pregnant women; levels of adherence and its influence on the anthropometric development of the newborn	Interview after birth, self-administered shorter Spanish version of “Kidmed” questionnaire, and clinical history review of mothers and newborns	492	85%	Peraita-Costa et al., 2018 [24]
Spain	To investigate maternal Mediterranean diet pattern adherence during pregnancy and its association with small-for-gestational-age and preterm birth	Two-phase retrospective population-based study of maternal dietary habits during pregnancy and their effect on newborn size and prematurity. Adherence to the Mediterranean diet assessed with the Spanish version of the Kidmed index	1118	77.3%	Peraita-Costa et al., 2021 [19]
Spain	To investigate the adherence to the Mediterranean diet of pregnant women from the NELA cohort	Food intake collected at 20 weeks of gestation using a validated FFQ	738	54%	Suárez-Martínez et al., 2021 [22]
Spain	To investigate the adherence to the Mediterranean diet and its association with newborn weight	A retrospective cross-sectional study. Anthropometric characteristics assessed at the beginning and the end of pregnancy; the other variables were assessed through a MEDAS questionnaire	218	NI/NA	Tomaino et al., 2020 [25]
Spain	To investigate the impact of the Mediterranean diet on pregnant women’s weight gain and obesity	A cross-sectional study	170	NI/NA	Silva del Valle et al., 2013 [20]
Spain	To research characteristics of maternal diet and lifestyle in early pregnancy and its association with GDM development	Questionnaire on nutritional knowledge, lifestyle, and dietary habits. Biochemical, obstetrics, and perinatal data monitored during pregnancy	103	NI/NA	Yuste-Gómez et al., 2022 [39]
Tanzania	To explore the micronutrient knowledge and dietary practices of Maasai pregnant women	Mixed-method study. Validated questionnaire and focus group	140	100%	Mshanga et al., 2020 [60]
Turkey	To evaluate the effect of pregnant nutrition education on nutritional knowledge levels	Nutrition education intervention. Face-to-face interviews for demographics and sources of nutritional knowledge. Nutritional knowledge questionnaire (25 items)	743	NI/NA	Aktaç et al., 2018 [67]
Turkey	To assess pregnant nutritional habits and healthy nutrition knowledge levels	General knowledge nutrition questionnaire	338	NI/NA	Aynaci et al., 2019 [66]
Uganda	To explore the maternal nutrition education offered by midwives to women attending an antenatal clinic	Six in-depth interviews with midwives, observation of six group education sessions, and twelve one-on-one interactions between midwives and pregnant women	6	NI/NA	Nankumbi et al., 2018 [74]
UK	To explore whether a Mediterranean diet reduces adverse pregnancy outcomes in high-risk women	A multicenter randomized trial. Intervention	1252 (593 IG/612 CG)	NI/NA	Al Wattar et al., 2019 [37]
UK	To explore women’s physical activity levels, diet, and gestational weight gain, and their experiences and motivations of behavior change	Cross-sectional data collected during a longitudinal cohort study. Dietary instrument for nutrition education (brief version), the International Physical Activity Questionnaire (short form), and open questions on perceptions of behavior change	193	NI/NA	Swift et al., 2017 [43]
USA	To investigate the effectiveness of an educational intervention on pregnant women’s nutritional knowledge	A quasi-experimental study. Intervention. Pre- and post-nutrition knowledge questionnaire (42 items)	27	NI/NA	Blondin et al., 2018 [42]
USA	To investigate pregnant women’s knowledge about gestational weight gain and how healthy eating behaviors impact it	Semi-structured individual interviews and focus group interviews	30	79%	Downs et al., 2014 [65]
USA	To explore the association between Mediterranean diet patterns around the time of conception and a lower risk of adverse pregnancy outcomes	Prospective, multicenter, cohort study. Diet assessment through FFQ	7798	NI/NA	Makarem et al., 2022 [35]
USA	To explore individual viewpoints of pregnant adolescents to facilitate the development of a nutrition intervention	A qualitative study using focus groups	14	NI/NA	Wise et al., 2015 [44]

Sample size refers to the number of subjects that participated in the study. NI/NA refers to “Not indicated” in the study or “Not applicable”, considering the design of the study. BMI: Body Mass Index. CG: Control Group. FFQ: Food Frequency Questionnaire. GDM: Gestational Diabetes Mellitus. HBM: Health Belief Model. IG: Intervention Group. Kidmed: Mediterranean Diet Quality Index for kids and adolescents. MEDAS: Mediterranean Diet Adherence Screener. MedDiet: Mediterranean Diet. MEDI-LITE: Literature-Based Adherence Score to Mediterranean Diet. NELA: Nutrition in Early Life and Asthma. UK: United Kingdom. USA: United States of America.

## 4. Conclusions

Despite the evidence supporting the importance of nutrition in the health of mother and fetus, the habits of pregnant women in different countries are far from the Mediterranean dietary pattern, which is considered a reference point in terms of dietary quality.

Pregnant women considered that one of the main barriers they encountered when implementing healthy habits was the lack of adequate advice from health professionals. They indicated that the information they received during pregnancy follow-up visits was too general, impractical, and insufficient. However, the success of nutritional education interventions carried out in different countries raised hopes for a well-structured, formal, nutritional education throughout pregnancy. It was likely that the origin of the lack of knowledge of pregnant women on certain food and nutrition issues was related to poorly planned nutrition education on the part of the responsible healthcare providers. Research on the consultations of those healthcare providers could be of help in proposing strategies to improve the content, depth, and duration of nutrition education sessions.

Finally, the limited nature of the available evidence in this field, which is scattered throughout the world, should be noted. Although the habits of pregnant women have been extensively studied, the relationship between those habits and the knowledge of pregnant women and the nutrition education they receive has been limited to certain geographical areas (African and Asian countries, Australia, Brazil, and the USA). The evidence available in Europe is limited to the Czech Republic, France, Norway, Romania, and the United Kingdom. Given the heterogeneity and lack of validation of some of the scales used to measure habits and knowledge in the available studies, any extrapolation of results between countries is complicated. Therefore, research that extends the evidence to different countries will be of help in understanding the factors that condition the habits of pregnant women and will therefore help guide strategies to improve the health of both mother and child during that all-important stage of their lives.

## Data Availability

No new data were created in this study.
